# Prospective analysis of BKV hemorrhagic cystitis in children and adolescents undergoing hematopoietic cell transplantation

**DOI:** 10.1007/s00277-021-04454-7

**Published:** 2021-03-04

**Authors:** Małgorzata Salamonowicz-Bodzioch, Jowita Frączkiewicz, Krzysztof Czyżewski, Olga Zając-Spychała, Ewa Gorczyńska, Anna Panasiuk, Marek Ussowicz, Krzysztof Kałwak, Zofia Szmit, Grażyna Wróbel, Bernarda Kazanowska, Alicja Chybicka, Bogna Ukielska-Hoffmann, Danuta Wendycz-Domalewska, Mariusz Wysocki, Magdalena Dziedzic, Jacek Wachowiak, Agnieszka Zaucha-Prażmo, Jerzy Kowalczyk, Jolanta Goździk, Jan Styczyński

**Affiliations:** 1 Department of Pediatric Stem Cell Transplantation, Hematology and Oncology, Medical University, ul. Borowska 213, 50-556 Wrocław, Poland; 2 Department of Pediatric Hematology and Oncology, Collegium Medicum, Nicolaus Copernicus University Torun, Bydgoszcz, Poland; 3 Department of Pediatric Oncology, Hematology and Transplantology, University of Medical Sciences, Poznan, Poland; 4 Central University Hospital, Wrocław, Poland; 5 Department of Pediatric Hematology, Oncology and Stem Cell Transplantation, Medical University, Lublin, Poland; 6 Stem Cell Transplant Center, University Children’s Hospital, Department of Clinical Immunology and Transplantology, Jagiellonian University Collegium Medicum, Krakow, Poland

**Keywords:** Papilloma viruses, BK virus, Hemorrhagic cystitis, Hematopoietic cell transplantation, Children

## Abstract

BK virus is one of the most common causes of hemorrhagic cystitis (HC) in children undergoing hematopoietic cell transplantation (HCT). Viruses can be found in urine and serum samples of immunocompromised patients. Malignant diseases, age, cell source, day of granulocyte reconstitution, conditioning regimen, or use of total body irradiation may play an important role in BKV epidemiology, development of hemorrhagic cystitis course, and outcome. The aim of this study was to evaluate the incidence, clinical course, and risk factors for BKV-HC in children undergoing HCT. A total number of 133 patients who were prospectively tested for BKV colonization/infection were enrolled into this multicenter analysis. Episodes of BKV-HC occurred in 36/133 (27%) enrolled subjects. In a univariate analysis for BKV-HC incidence, the following factors were significant: age >5 years, peripheral blood transplantation, matched unrelated donor (MUD) transplantation, busulfan-cyclophosphamide-melphalan conditioning regimen, and acute myeloblastic leukemia (AML) diagnosis. Presence of acute graft-versus-host disease (aGVHD) in liver and gut GVHD was a significant risk factor of BKV-HC. No BKV-attributed deaths were reported. In multivariate analysis, the incidence of HC was significantly higher in patients with AML, age >5 years, MUD transplants, and children with GVHD. HC is a frequent complication after HCT among children causes prolonged hospitalization but rarely contributes to death. We identified risk factors of BKV-HC development in children, with focus on aGVHD: we concluded that excessive immune reaction connected with GVHD and immunosuppression drugs might play a pivotal role in the development of BKV-HC.

## Introduction

Human polyomavirus BKV (Polyomavirus hominis 1) was first identified in 1970 from the urine of a renal allograft of a Sudanese recipient named BK, who developed stenosis of ureter [1]. The virus belongs to the *Polyomaviridae* family, DNA viruses containing circular double-stranded DNA. More than 80% of the adult population is exposed during life to BK virus [2–4]. Primary BKV infection is mostly asymptomatic. After primary infection, the virus remains dormant in the urinary tract, lymphoid tissues, and leucocytes. BKV can cause neoplastic transformation [5], pneumonia [6], graft nephropathy in renal allograft [7], and hemorrhagic cystitis (HC) in people after hematopoietic cell transplantation (HCT). BK viruria has been associated with a variety of clinical manifestations in HCT recipients, including asymptomatic hematuria, hemorrhagic cystitis [2, 3, 8], stenosis of the ureter, and interstitial nephritis. HC is associated with significantly higher morbidity and mortality among adult recipients [2, 9, 10]. Post-engraftment HC is very rare among patients who undergo autologous as compared with allogeneic HCT, even though similar myeloablative conditioning regimens are used. It underlines that alloimmune reactions after HCT can play an important role in the development of infection. Hence, majority of HC cases occur in allogeneic HCT with GVHD [11, 12].

The risk factors for BKV reactivation following HCT include haploidentical or cord blood transplantation, acute graft-versus-host disease (aGVHD), and coinfection with cytomegalovirus (CMV) [9, 13, 14]. Apart from that, malignant diseases, age, sex, cell source, day of granulocyte reconstitution, conditioning regimen, or use of total body irradiation (TBI) may play an important role in BKV course. BK subtypes and the BK urine viral load have been identified as additional risk factors for BK virus–associated with incidence and course of hemorrhagic cystitis [14–16].

The therapeutic approach depends on the severity and dynamics of the disease. The current standard of care of BK virus infection includes analgesia, hyper-hydration, forced diuresis, and continuous bladder irrigation to avoid renal obstruction and clot formation [8]. It is important to maintain platelets above 50 G/L and a hematocrit over 25%. Non-severe cases of BK virus–associated HC usually resolve spontaneously over 2 weeks with supportive care only. In severe cases with significant bleeding and urinary tract obstruction, cystoscopy and catheterization must be performed to preserve the function of the kidneys. In profuse bleeding, and life-threatening conditions, surgical intervention, like cystectomy, must be considered [17, 18]. Some clinicians use topical agents (alum, formalin) and prostaglandin E1, which are installed in the urine bladder. These methods somehow remain controversial and not clinically proven [18]. There is no licensed antiviral drug active against BKV. However, several drugs are used in clinical practice.

Cidofovir (CDV), which is an acyclic nucleoside analog with anti-polyomavirus activity, has been demonstrated in in vitro studies to have activity against the BK virus [19–22]. Cidofovir is originally licensed for the treatment of CMV retinitis in AIDS patients and as a second-line drug for the treatment of ganciclovir-resistant CMV infections. Cidofovir treatment results in a sustained suppression of CMV replication and a significant reduction of BK viruria [23]. Intravesicular treatment for BKV-HC with cidofovir seems to be a promising method. Drug is used at a dosage of 5 mg/kg per instillation. When administered 2–7 times, it seems to be effective as 59% of patients demonstrated complete clinical resolution of symptoms; 28% had a partial response; and 13% had no change in symptoms. Patients with a high HC grade and high pre-treatment BK viral load (>100 million copies/mL) had a lower frequency of complete remission. The main adverse effects of intravesicular instillation were severe bladder spasms [23]. Apart from CDV, also calcineurin inhibitors, quinolone antibiotics, nalidixic acid, and oxolinic acid can inhibit BK virus replication in vitro [24–27].

The aim of this multicenter prospective study was to determine the incidence, clinical course, and outcome of BKV-HC in pediatric patients after allogeneic HCT.

## Materials and methods

### Design of the study

Pediatric patients (aged 1–18 years), transplanted between January 2018 and December 2019, were enrolled into the prospective study of monitoring for BKV before and after allo-HCT and followed up for a period of a minimum of 6 months or death, whichever occurred first. Children were transplanted in all 5 pediatric transplant centers in Poland (Wrocław, Bydgoszcz, Poznan, Lublin, and Krakow). The centers did not differ from each other in terms of standard of care, type of conditioning regimens, and approach to BKV management. Routine BKV testing is not a standard of care in HCT setting in our country. Pediatric patients undergoing allo-HCT, who have been screened for BKV, were included in the study. Patients were excluded if they have been PCR-positive for CMV, ADV, and EBV at the time of BKV testing and have been actively treated due to another viral infection.

Patients were divided into 3 subgroups: BKV1, BKV2, and BKV3. BKV1 group (control group) included patients BKV-negative, without symptoms of BKV-HC. BKV2 patients are those with BKV-positive sample (urine and/or serum) without symptoms of BKV-HC, who did not require any directed treatment. BKV3 patients are those with symptoms of BKV-HC and positive urine or blood samples. Treatment with antiviral drugs and symptomatic treatment was administered to patients in this group, including surgical/urological intervention if necessary.

Upon inclusion, a written informed consent was obtained from all parents of participants. The inclusions were made by a transplant physician and a medical history including review of the medical records was reported. The dataset including age, gender, clinical findings, laboratory results, and predisposing factors was obtained. The inclusion criteria in the control group were solely BKV-negative result in HCT subjects and the two exclusion criteria were (1) positive result for CMV, ADV, and EBV, and (2) treatment due to these viral infections. The study was approved by the Bioethical Committee in Bydgoszcz (no. KB 499/2014).

### Conditioning regimens

Patients were treated with reduced toxicity regimens (RTC), reduced intensity protocol (RIC), and myeloablative conditioning (MAC). Reduced toxicity conditioning (RTC) consisted mainly of fludarabine (160 mg/m^2^) and treosulfan (36–42 mg/kg) with thiotepa (10 mg/kg), cyclophosphamide (120 mg/kg), or melphalan (140 mg/m^2^). In RIC regimen, fludarabine with either melphalan or low non-myeloablative doses of busulfan (2 mg/kg) was used. MAC was based either on myeloablative doses (12.8–19.6 mg/kg) of busulfan or on total body irradiation (TBI) at a median dose of 12 Gy. Type of conditioning, donor, graft source, and reconstitution day are presented in Table 1.

**Table 1:** Characteristics of patients

Total number of patients	133
Sex	
Male	85 (63.9%)
Female	48 (36.1%)
Age	
>5 years	97 (72.9%)
<5 years	36 (27.1%)
Diagnosis	
Acute lymphoblastic leukemia (ALL)	57 (42.9%)
Acute myeloblastic leukemia (AML)	11 (8.3%)
Severe aplastic anemia (SAA)	19 (14.3%)
Myelodysplastic syndromes (MDS)	10 (7.5%)
Primary immunodeficiencies (PID)	12 (9.0%)
Other	24 (18.0%)
Conditioning regimens	
TBI-VP	21 (15.8%)
TREO-FLU-TT	40 (30.1%)
BU-FLU-TT	10 (7.5%)
BU-CY-MEL	11 (8.3%)
CY-FLU	12 (9.0%)
Other	38 (28.5%)
Missing	1 (0.80%)
BKV colonization	16 (12%)
Donor	
Matched unrelated donor (MUD)	89 (66.9%)
Matched sibling donor (MSD)	38 (28.6%)
Mismatched relative (MMREL)	6 (4.5%)
Neutrophil recovery	
Day ≤15 after HCT	70 (52.6%)
Day >15 after HCT	62 (46.6%)
Missing	1 (0.8%)

*TBI-VP*, total body irradiation-etoposide; *TREO-FLU-TT*, Treosulfan-fludarabine-thiotepa; *BU-FLU-TT*, busulfan-fludarabine-thiotepa; *BU-CY-MEL*, busulfan-cyclophosphamide-melphalan; *CY-FLU*, cyclophosphamide-fludarabine

As a standard GVHD prophylaxis, all patients, since 1 day before HCT, received intravenous cyclosporine (CsA) in a dose of 1.5 mg/kg twice per day in 2-h infusions. Further dosage of CsA was adjusted according to the CsA level (target level: 100–200 μg/L), monitored twice a week in the majority of patients, and then switched into oral formulation (in stable patients who were able to accept oral intake). According to standard protocols, in patients without GVHD signs and symptoms, CsA administration was continued to day 120, preceded by slow tapering. The second prophylactic drug, methotrexate (MTX), was administered three times, in a standard dose of 10 mg/m^2^ on days 1, 3, and 6 after HCT. All patients transplanted from alternative donors or treated for severe aplastic anemia were given in vivo T-cell depletion by either rabbit anti-thymocyte globulin (ATG) (ATG-Fresenius/Grafalon®) at a median dose of 45 mg/kg or Thymoglobuline® at a median dose of 7.5 mg/kg, or Campath-1H at median dose 1 mg/kg.

### Prophylaxis of infections

All enrolled patients were followed starting from the day of transplantation up to at least 100 days post transplantation. HCT patients were hospitalized in single-bed rooms. Antimicrobial prophylaxis consisted of oral colistin or rifaximin from the beginning of the conditioning regimen. In BKV-positive patients aged >4-year oral ciprofloxacin was administered in the majority of centers. Younger or BKV-negative subjects were not on quinolone prophylaxis. Oral or intravenous acyclovir and oral posaconazole were started at the onset of the conditioning regimen. Prophylactic trimethoprim/sulfamethoxazole was given to all patients before and after HCT until at least one month after the end of immunosuppression. Antifungal prophylaxis with posaconazole was continued until the end of immunosuppressive therapy. First-line intravenous antibiotic empiric therapy usually included a broad-spectrum beta-lactam and aminoglycoside.

### Diagnosis of BK virus

Quantitative BKV viruria and BKV viremia were checked once a week (every 7 days) by PCR detection of viral DNA in all transplanted patients starting from a day of admission to the department. In patients having dysuria, oliguria, anuria or hematuria, and/or hemorrhage, serum and urine samples were tested each time in case of symptoms. Asymptomatic patients were screened for 100 days after HCT. Patients presenting symptoms were checked until the resolution of BKV-HC signs or virus negativity, whichever occurred first. Additionally, all asymptomatic HCT patients were screened weekly for the presence of other viruses (adenovirus, cytomegalovirus, Epstein-Barr virus, rotavirus), *Clostridioides difficile*, and multi-resistant bacteria, as a part of routine monitoring.

Colonization with BKV was defined as having virus in urine/blood at a moment of admission to the transplant ward before the start of conditioning regimen. BKV viruria was defined if ≥500 copies/mL were detected by PCR at any time point. High BKV viruria was defined if ≥1×10^7^ copies/mL were detected. BKV hemorrhagic cystitis (BKV-HC) was defined by presence of some of the following symptoms like dysuria, hematuria, oliguria, or urinary tract hemorrhage in a patient with presence of BKV in blood and/or urine, and respective treatment was needed. Time to BKV infection was defined as a time from first day of transplant to BKV-HC episode (virus found in blood and/or urine) and presence of symptoms.

### Treatment of BK

In case of the absence of BKV, any particular prophylaxis was not used (control group, BKV1). In case of presence of BK virus in serum or urine without clinical symptoms (colonization, BKV2 group) before high-dose chemotherapy, prophylaxis with oral ciprofloxacin (2 × 10 mg/kg) was used in children aged >4 years. In case of presence virus in serum and urine and clinical manifestation (BKV3 group), intravenous cidofovir (5 mg/kg) with probenecid (3+1 g or 1.5+0.5 g orally depending on body weight and/or age) was used once a week until absence of clinical symptoms or virus negativity, whichever occurred first. Intravesicular cidofovir at the dose of 5 mg/kg/week was used in case of very severe HC, where intravenous cidofovir was not efficient enough (presence of symptoms of BKV-HC and high BKV viruria or/and viremia) after 5 doses of CDV or profuse nephrotoxicity. Apart from that, supportive treatment as analgesia, hyper-hydration, forced diuresis, and continuous bladder irrigation was administered. Surgical intervention was used only in severe cases with significant bleeding and urinary tract obstruction to preserve renal function.

### Statistical analysis

To compare differences between groups, the chi-square test or Fisher exact test was used for categorical variables and the Mann-Whitney *U* test for continuous variables. Odds ratio (OR) and 95% confidence intervals (95%CI) are shown. The cumulative incidences of BKV infection were assessed using competing risk analysis and Gray’s test. A multivariate logistic regression using the stepwise model selection method was used to evaluate potential risk factors that might influence donor outcome variables. The following risk factors were analyzed: age, primary diagnosis, conditioning, type of donor, HLA match, cell source, neutrophil engraftment, presence of GVHD, BKV colonization, and presence of BKV in blood ± urine. *p*<0.05 was regarded as significant.

## Results

### Demographics

A total number of 133 allo-HCT patients (BKV1+BKV2+BKV3 groups), including 48 girls and 85 boys, were included in the study, with a median age of 8.5 (range 0.3–19.9) years. Children were transplanted due to acute lymphoblastic leukemia (ALL; *n*=57), acute myeloblastic leukemia (AML; *n*=11), myelodysplastic syndromes (MDS; *n*=10), severe aplastic anemia (SAA; *n*=19), primary immunodeficiencies (PID; *n*=12), Nijmegen Breakage Syndrome (NBS; *n*=3) juvenile myelomonocytic leukemia (JMML; *n*=4), chronic myeloid leukemia (CML; *n*=3), or other diseases (*n*=14) (Table 1).

### Incidence of BKV

#### BKV2 group

46 patients (35%) including 30 boys and 16 girls with positive BKV results were asymptomatic. The median age was 8.6 years (range 0.5–19 years). Previous colonization with BKV was found in 12 of them (26%). Patient distribution for colonization and infection is shown in Table 2.

**Table 2:** Incidence of BKV

Diagnosis	BKV-1	BKV-2	BKV1 + BKV2	BKV-3	P (BKV3 vs BKV1/2)
Total	51 (100%)	46 (100%)	97 (100%)	36 (100%)	
ALL	25 (49.0%)	19 (41.3%)	44 (45.4%)	13 (36.1%)	0.44
AML	2 (3.9%)	2 (4.3%)	4 (4.1%)	7 (19.4%)	0.012
SAA	7 (13.7%)	10 (21.7%)	17 (17.5%)	2 (5.6%)	0.14
MDS	1 (2.0%)	6 (13.0%)	7 (7.2%)	3 (8.3%)	0.99
PID	10 (19.6%)	1 (2.2%)	11 (11.3%)	1 (2.8%)	0.23
Neutrophil recovery					
Day ≤15 after HCT	17 (33.3%)	28 (60.9%)	45 (46.4%)	17 (48.6%)	0.98
Day >15 after HCT	34 (66.7%)	18 (39.1%)	52 (53.6%)	18 (51.4%)	
Source					
BM	34 (66.7%)	11 (23.9%)	45 (46.4%)	5 (13.9%)	0.0012
PB	17 (33.3%)	35 (76.1%)	52 (53.6%)	31 (86.1%)	
Donor					
MUD	27 (52.9%)	29 (63.0%)	56 (57.7%)	33 (91.7%)	0.0005
MSD	21 (41.2%)	15 (32.6%)	36 (37.1%)	2 (5.6%)	
T-cell depletion in vivo					
ATG/anti-CD52	32 (62.7%)	37 (80.4%)	69 (71.1%)	34 (94.4%)	0.008
No serotherapy	19 (37.3%)	9 (19.6%)	28 (28.9%)	2 (5.6%)	
Conditioning regimen	51 (100%)	45 (100%)	96 (100%)	36 (100%)	
None	-	2 (4.4%)	2 (2.1%)	-	0.94
TBI-VP	12 (23.5%)	5 (11.1%)	17 (17.7%)	4 (11.1%)	0.51
TREO-TT	14 (27.5%)	17 (37.8%)	31 (32.3%)	9 (25.0%)	0.54
BU-FLU-TT	2 (3.9%)	4 (8.9%)	6 (6.3%)	4 (11.1%)	0.56
BU-CY-MEL	1 (2.0%)	4 (8.9%)	5 (5.2%)	6 (16.7%)	0.077
CY-FLU	1 (2.0%)	10 (22.2%)	11 (11.5%)	1 (2.8%)	0.22
TREO-FLU	5 (9.8%)	-	5 (5.2%)	-	0.37
Other	16 (31.4%)	3 (6.7%)	19 (19.8%)	12 (33.3%)	0.16
GVHD before BKV infection					
No	47 (92.2%)	26 (56.5%)	73 (75.3%)	16 (44.4%)	0.0015
Yes	4 (7.8%)	20 (43.5%)	24 (24.7%)	20 (55.6%)	
GVHD					
Absent	39 (76.5%)	25 (54.3%)	64 (66.0%)	14 (38.9%)	0.005
Acute	11 (21.6%)	17 (37.0%)	28 (28.9%)	18 (50.0%)	0.026
Acute + chronic	1 (2.0%)	3 (6.5%)	4 (4.1%)	3 (8.3%)	0.39
Chronic		1 (2.2%)	1 (1.0%)	1 (2.8%)	0.99
Acute GVHD					
No	39 (76.5%)	26 (56.5%)	65 (67.0%)	15 (41.7%)	0.009
Yes	12 (23.5%)	20 (43.5%)	32 (33.0%)	21 (58.3%)	
Chronic GVHD					
No	50 (98.0%)	42 (91.3%)	92 (94.8%)	32 (88.9%)	0.25
Yes	1 (2.0%)	4 (8.7%)	5 (5.2%)	4 (11.1%)	
GVHD localization					
Absent	39 (76.5%)	25 (54.3%)	64 (66.0%)	13 (36.1%)	0.003
Skin	11 (21.6%)	13 (28.3%)	24 (24.7%)	11 (30.6%)	0.64
Gut ± skin	1 (2.0%)	5 (10.9%)	6 (6.2%)	7 (19.4%)	0.042
Liver ± others		3 (6.5%)	3 (3.1%)	5 (13.9%)	0.033
GVHD: gut + ski**n**					
No	50 (98.0%)	41 (89.1%)	91 (93.8%)	29 (80.6%)	0.042
Yes	1 (2.0%)	5 (10.9%)	6 (6.2%)	7 (19.4%)	
GVHD: liver					
No	51 (100%)	43 (93.5%)	94 (96.9%)	31 (86.1%)	0.033
Yes	-	3 (6.5%)	3 (3.1%)	5 (13.9%)	
GVHD: skin + liver					
No	50 (98.0%)	38 (82.6%)	88 (90.7%)	24 (66.7%)	0.002
Yes	1 (2.0%)	8 (17.4%)	9 (9.3%)	12 (33.3%)	
GVHD stadium					
0	39 (76.5%)	25 (54.3%)	64 (66.0%)	13 (36.1%)	0.002
1	4 (7.8%)	-	4 (4.1%)	1 (2.8%)	0.99
2	6 (11.8%)	10 (21.7%)	16 (16.5%)	10 (27.8%)	0.15
3	2 (3.9%)	6 (13.0%)	8 (8.2%)	7 (19.4%)	0.12
4	-	5 (10.9%)	5 (5.2%)	5 (13.9%)	0.13
GVHD-3–4					
No	49 (96.1%)	35 (76.1%)	84 (86.6%)	25 (69.4%)	0.039
Yes	2 (3.9%)	11 (23.9%)	13 (13.4%)	11 (30.6%)	

*TBI-VP*, total body irradiation-etoposide; *TREO-FLU-TT*, treosulfan-fludarabine-thiotepa; *BU-FLU-TT*, busulfan-fludarabine-thiotepa; *BU-CY-MEL*, busulfan-cyclophosphamide-melphalan; *ALL*, acute lymphoblastic leukemia; *AML*, acute myeloid leukemia; *SAA*, severe aplastic anemia; *MDS*, myelodysplastic syndromes; *PID*, primary immunodeficiencies; *MSD*, matched sibling donor; *MUD*, matched unrelated donor; *PB*, peripheral blood; *BM*, bone marrow; *GVHD* graft-versus-host disease, *ATG* anti-thymocyte globulin

#### BKV3 group

The incidence of BKV infection (BKV-HC; BKV3) was 27% (36/133 cases). Among children who developed HC symptoms, there were 13 girls and 23 boys, at median age 10.7 (range 0.3–19.9) years. BKV was isolated from blood in 7 cases (19%), from urine in 19 cases (53%), and from both in 10 cases (28%) (Table 2). Previous colonization with BKV was found in 4 children (11%). Diagnosis distribution is presented in Tables 1 and 2. Overall 23/36 patients with BKV-HC (64%) developed graft-versus-host disease (GVHD). Eighteen of them (78%) suffered from acute GVHD (aGVHD), and 4 patients (17%) developed chronic or acute/chronic GVHD. Detailed information concerning GVHD grade and location are presented in Table 2.

#### BKV2+BKV3 groups

**Fig. 1:**
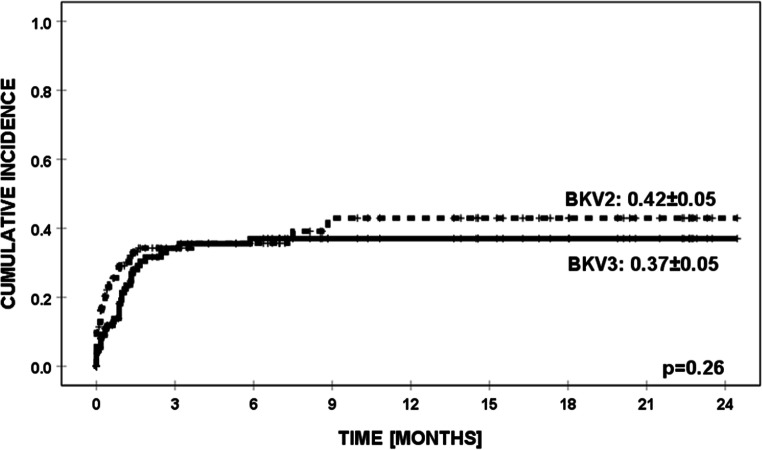
Cumulative incidence of BKV in BKV2 and BKV3 groups

BKV-positive results (urine and/or blood) were observed in 82 patients (62%), including 53 boys and 29 girls (median age was 9.65 years; range 0.3–19.9 years). The site of isolation and type of diagnosis are presented in Tables 1 and 2. Cumulative incidence of BKV infection in BKV2 and BKV3 groups is presented in Fig. 1.

### Clinical course/manifestation of BKV

Overall, 36 patients (27%) developed signs and symptoms of HC: dysuria, bleeding from urinary tract, and problems with passing urine or and anuria. Few patients, apart from HC symptoms presented with fever, cough, or diarrhea. The median time to develop BKV infection was 27 days (range 0–177 days) from the beginning of conditioning regimen.

### Treatment

In the BKV3 group, treatment with weekly CDV was applied in 98.8% of cases, and with foscarnet in one case. The median number of CDV doses was 3 (range: 1–5). The mean period of treatment was 28 days (range: 5–132). In 3 patients, intravesicular CDV was additionally administered due to persistent symptoms and a high grade of HC or nephrotoxicity. In 2 cases, due to significant bleeding and urinary tract obstruction, cystoscopy and catheterization were necessary. Anti-BKV treatment caused the resolution of symptoms in all cases. There were no recurrences of BKV-HC. One patient developed renal failure due to urinary tract obstruction caused by bleeding clots.

### Deaths

Overall, 13 patients died, including 10 with BKV-HCs; however, no death was attributed to BKV infection. In 7/13 cases, the death was secondary to generalized inflammatory response syndrome with multi-organ failure caused by other infections (bacterial, fungal). The primary cause of other deaths was progression of underling disease (*n*=4), GVHD (*n*=1), and others (*n*=1).

### Risk factors analysis for BKV incidence

In univariate analysis, significantly lower incidence of BK-HC was found in children after bone marrow transplantation (OR 0.18; 95%CI 0.06–0.50; *p*=0.001), and MSD transplant (OR 0.10; 95%CI 0.02–0.44; *p*=0.002). A higher incidence of BKV-HC was observed in the case of the following: peripheral blood transplantation (OR 5.36; 95%CI 1.92–14.96; *p*=0.001), matched unrelated donor (MUD) transplantation (OR 8.05; 95%CI 2.31–28.07; *p*=0.001), having T-cell depletion in vivo by the use of serotherapy with ATG/alemtuzumab (OR 6.90; 95%CI 1.60–30.7; *p*=0.011), using BU-CY-MEL conditioning regimen (OR 3.64; 95%CI 1.03–12.78; *p*=0.04), age >5 years (OR 5.67; 95%CI 1.61–19.8; *p*=0.007), and diagnosis of AML (OR 5.61; 95%CI 1.53–20.53; *p*=0.009). Patient with aGVHD (OR 2.84; 95%CI 1.29–6.24; *p*=0.009), including liver GVHD (OR 5.05; 95%CI 1.14–22.37; *p*=0.03) and gut GVHD (OR 3.66; 95%CI 1.13–11.76; *p*=0.029), had higher incidence of BK-HC. High grade of GVHD influenced significantly the incidence of BKV-HC. Patients with grade III–IV GVHD had a higher incidence of BK-HC (OR 2.84; 95%CI 1.13–7.12; *p*=0.026). There was no significant impact of reconstitution day, HLA matching, sex, previous urine colonization, site of colonization (urine vs blood) on incidence of BKV hemorrhagic cystitis (Table 3).

**Table 3:** Univariate analysis of risk factors for BKV-HC

Risk factors	OR (95%CI)	*p* value
Age >5 years	5.7 (1.6–19.8)	0.007
Sex: male vs female	0.99 (0.4–2.2)	0.998
Conditioning regimen		
TBI-VP	0.6 (0.2–1.8)	0.36
TREO-FLU-TT	0.7 (0.3–1.6)	0.418
BU-FLU-TT	1.9 (0.5–7.1)	0.354
BU-CY-MEL	3.6 (1.1–12.7)	0.044
CY-FLU	0.2 (0.02–1.7)	0.156
Diagnosis		
ALL	0.7 (0.3–1.5)	0.339
AML	5.6 (1.5–20.5)	0.009
SAA	0.3 (0.06–1.2)	0.097
MDS	1.2 (0.3–4.8)	0.828
PID	0.2 (0.03–1.8)	0.159
MSD	0.1 (0.02–0.4)	0.002
MUD	8.1 (2.3–28)	0.001
HLA match	0.5 (0.2–1.4)	0.208
PB vs BM	5.3 (1.9–14.9)	0.001
T-cell depletion in vivo	6.9 (1.6–30.7)	0.011
Neutrophil recovery >15 day	0.9 (0.4–1.9)	0.825
GVHD		
GVHD before BKV infection	3.8 (1.7–8.4)	0.001
Acute GVHD	2.8 (1.3–6.2)	0.009
Chronic GVHD	2.3 (0.6–9.1)	0.235
GVHD gut + skin	3.6 (1.1–11.7)	0.029
GVHD liver	5.1 (1.1–22.3)	0.033
GVHD skin + liver	4.9 (1.8–12.9)	0.001
GVHD grades 3–4	2.8 (1.1–7.1)	0.026
Site of BKV isolation		
Colonization	0.8 (0.2–2.9)	0.843
BKV viremia	1.6 (0.5–5.3)	0.434
BKV viruria	1.0 (0.4–2.4)	0.957
BKV urine + plasma	0.7 (0.3–1.8)	0.499

*TBI-VP*, total body irradiation-etoposide; *TREO-FLU-TT*, treosulfan-fludarabine-thiotepa; *BU-FLU-TT*, busulfan-fludarabine-thiotepa; *BU-CY-MEL*, busulfan-cyclophosphamide-melphalan; *ALL*, acute lymphoblastic leukemia; *AML*, acute myeloid leukemia; *SAA*, severe aplastic anemia; *MDS*, myelodysplastic syndromes; *PID*, primary immunodeficiencies; *MSD*, matched sibling donor; *MUD*, matched unrelated donor; *PB*, peripheral blood; BM, bone marrow; *GVHD*, graft-versus-host disease

In multivariate analysis, factors significantly contributing to higher incidence of BKV-HC were as follows: AML diagnosis (OR 9.57; 95%CI 1.85–49.2; *p*=0.007), age >5 years (OR 10.78; 95%CI 2.26–51.43; *p*=0.003), MUD transplants (OR 6.25; 95%CI 1.38–28.2; *p*=0.017), and presence of GVHD before BKV infection (OR 2.82; 95%CI 1.08–7.34; *p*=0.033). No impact of conditioning regimen, T-cell depletion in vivo, and stem cell source on incidence of BKV-HC was found (Table 4).

**Table 4:** Multivariate analysis of risk factors for BKV-HC

Risk factors	OR (95%CI)	*p* value
Age: >5 vs <5 years	10.8 (2.3–51.4)	0.003
Conditioning: BU-CY-MEL vs other	3.4 (0.5–22.2)	0.208
Diagnosis: AML vs other	9.6 (1.9–49.3)	0.007
Donor: MUD vs MSD	6.3 (1.4–28.3)	0.017
T-cell depletion in vivo	1.9 (0.1–28.6)	0.629
Cell source: PB vs BM	3.2 (0.96–10.7)	0.057
GVHD before BKV infection: yes vs no	2.8 (1.1–7.3)	0.033

*BU-CY-MEL*, busulfan-cyclophosphamide-melphalan; *AML*, acute myeloid leukemia; *MUD*, matched unrelated donor; *MSD*, matched sibling donor; *PB*, peripheral blood; *BM*, bone marrow; *GVHD*, graft-versus-host disease

## Discussion

We analyzed incidence and the clinical risk factors for HC after allo-HCT in pediatric population, with emphasis on factors contributing to the development and severity of HC. The HC incidence was 27% among enrolled patients, relatively higher in comparison to other studies [28–30].

Underling disease can play an important role in developing BKV-HC after HCT. Riachy et al. [31] has shown on univariate analysis that increased risk of hemorrhagic cystitis was significantly associated with underlying diagnoses of rhabdomyosarcoma, acute leukemia, and aplastic anemia. Mori et al. [32] highlighted that underlying disease like acute leukemia versus others is important risk factor for BKV-HC. In contrast, Silva et al. [33] observed that diagnosis did not play an important risk factor for BKV-HC. In our study, we have found that AML was a significant risk factor for developing HC in BKV-positive patients.

Another very important factor concerning the developing of BKV-HC is age. Regarding some retrospective studies describing pediatric cohorts in univariate analysis increased risk of hemorrhagic cystitis was significantly associated with age >5 years [31]. Among adult patients, after allo-HCT, no significant impact was found for age [33]. In our study, the incidence of BKV infection was significantly higher in patients >5 years both in univariate and multivariate analysis. It is probably caused by the fact that older patients used to get asymptomatic BKV infection in early childhood, which persisted latent in their bodies. On the other hand, we did not find the correlation between colonization with BKV and higher predisposition to BKV-HC.

Donor type seems to be an important risk factor causing hemorrhagic cystitis. Incidence of HC was higher in unrelated donor transplantation (MUD) [14, 30, 34]. El-Zimaity et al. presented that patients who received a graft from a related donor had the lowest rate of HC, followed by patients with a mismatched related donor, cord blood grafts, and MUD [35]. In our study, MUD transplants were significantly associated with higher incidence of BKV-HC. In contrast, Silva et al. presented cumulative incidence at about 16% in all transplanted groups. It was highest (58%) among patients after haploidentical ablative regimens and cord blood transplant and those who have positive BKV PCR before transplant [33].

The connection between HC and GVHD has been studied for many years, but still seems to be unclear [10, 36–38]. Other studies have presented GVHD as a risk factor for the BKV-HC, especially severe or late-onset HC [36, 39–41]. However, it still remains not clear whether GVHD targeting bladder epithelium is manifested as HC or whether immunosuppressive drugs used to treat acute GVHD as well as GVHD itself contributes to HC and its grade [42, 43]. Acute GVHD preceded HC in about 50% of the HC patients; however, the use of steroids and aGVHD was much more frequent at the onset of HC than at the resolution of HC. Bogdanovic et al. highlighted that the combination of BK virus together with aGVHD predicted the development of HC better than acute GVHD alone [14]. In our study of patients with aGVHD, especially the liver and gut, GVHD had a significantly higher incidence of BK-HC. High grade of GVHD (grades III–IV) influenced significant incidence of BKV-HC. It is not in line with Silva et al. [33] and El-Zimaity et al. [35] who did not observe that diagnosis of grade II–IV aGVHD had significant impact on developing HC. Contrary, Saade et al. [44] highlighted that occurrence of a grade II to IV acute GvHD was an important risk factor associated with the development of HC.

Conditioning regimen is a very important factor in development of hemorrhagic cystitis. Mechanism has been well described by many authors [45–47]. In our prospective study, we found that conditioning regimens which included both busulfan and cyclophosphamide increased the risk of HC. We indicate that cyclophosphamide used in RIC protocols was not significantly associated with HC compared to MAC (BY-CY-MEL) containing cyclophosphamide. This might have resulted from the higher dose of cyclophosphamide in MAC [48]. In some studies, there was no relation between dose of cyclophosphamide [30, 47] or interaction with TBI.

Stem cell source as a risk factor was not well described. Riachy et al. [31] on multivariate analysis have shown that allogeneic bone marrow or peripheral blood stem cell transplantation and pelvic radiotherapy were significantly associated with increased risk of hemorrhagic cystitis. Silva et al. [33] did not find a correlation between donor source and BKV-HC. In our univariate analysis, significantly higher incidence of BKV-HC was connected with peripheral blood transplantation than with bone marrow HCT.

HLA matching and neutrophil engraftment were not connected with higher incidence of BKV-HC in our prospective analysis. It was in line with other studies [33, 35], where similarly no significant impact was found for platelet and neutrophil engraftment.

Similarly to El-Zimaity et al. [35], the median onset of clinical symptoms in our study was 27 (range: 2–875) days. It was not consistent with previous reports [33, 37, 49–51], where the median time to develop symptoms was 49 (range: 2–287) days. The median duration time of treatment was 28 days (range 0–177 days) and a median of 3 doses of CDV was given. Other studies have presented similar results [52], but not all of them included pediatric population.

The influence of HC on the final outcome remains controversial. According to some studies, patients with BKV-HC had low probability of survival (<20%), but on the other hand, Gorczynska et al. [18] have reported that clinical course of BKV-HC was less severe than that caused by ADV. In that study, the main cause of deaths was not attributed to BKV infection but rather to the progression of the underlying disease. Similarly, we had 10 deaths among patients with BKV-HC but there was no death caused directly by BKV, although in majority of cases, death was secondary to generalized inflammatory response with MOF (often with liver failure) caused by progression of underlying disease, bacterial/fungal infections, or GVHD.

The limitation of the study was no unification in approach to fluoroquinolones prophylaxis in children below 4 years of life which differed between centers and screening for BKV. Another limitation was time to obtain results of BKV viremia (e.g., up to 1 day in some centers and up to 4–5 days in the others).

In conclusion, HC remains frequent and troublesome particularly in the clinically severe stage, often causing prolonged hospitalization but rarely contributed to death among children after HCT. BKV-HC occurred in 27% of enrolled prospectively analyzed children. We have identified different risk factors in HCT recipients of developing BKV-HC, although there are limitations to this analysis. The highest incidence occurred in patients with AML and age of >5 years. Myeloablative conditioning containing cyclophosphamide and busulfan presents a higher risk for HC than RIC. Children transplanted from an unrelated donor or with PBSCT are more predisposed to develop BKV-HC. Previous urine colonization (before HCT) and site of isolation of the virus (urine ± blood) after HCT did not influence the development of BKV-HC. Excessive immune reaction (connected with GVHD and used immunosuppressive drugs) might play a pivotal role for the development of BKV-HC. Acute GVHD has been shown to be an important risk factor for developing HC. Children having GVHD of gastrointestinal tract (liver or and gut), especially of high grade (III/IV) are at the highest risk of hemorrhagic cystitis. Umbro et al. [2, 53, 54] highlighted that in patients being on immunosuppression, BKV infection has been associated with other pathologies such as ureteral stenosis, vasculopathies, pneumonia, hepatitis, encephalitis, and multi-organ failure. We hypothesize that in some cases, BKV can affect the liver, leading to development of symptoms and signs (hypertransaminasemia, hyperbilirubinemia, liver failure) similar to that of liver GVHD and can be misdiagnosed as a liver GVHD treated with high immunosuppression but finally, it did not turn out to be a real GVHD. Liver biopsy should be always done in children with liver symptoms suspected for GVHD of liver and presence of BKV to avoid incorrect treatment which can be fatal for viral infection.
